# Mission Impossible: Densah Bur Revolution in Treating Atrophic Maxilla

**DOI:** 10.7759/cureus.77040

**Published:** 2025-01-06

**Authors:** Madhurya Remalli, Mounika V, Lakshmi Rathan A C, Vivek N, Prashanthi Gurram

**Affiliations:** 1 Department of Oral and Maxillofacial Surgery, SRM Kattankulathur Dental College and Hospital, SRM Institute of Science and Technology, Chengalpattu, IND

**Keywords:** atrophic maxilla, densah burs, dental implant surgery, oral rehabilitation, osseodensification

## Abstract

Atrophic maxilla is a condition that compromises the bone density of the jaws. The three-dimensional remodeling of bones makes it more difficult to plan rehabilitation. Bone density plays a key role in implant placement, but in the case of the atrophic maxilla, placement of an implant with conventional burs is difficult and often leads to a failed implant prosthesis. To overcome this complication, Densah burs emerged as a solution for such a challenging situation. In this article, we reported a case of a 55-year-old male patient presented with the posterior atrophic maxilla. Preoperative radiological assessment was done using cone beam computed tomography (CBCT) to evaluate the bone density. Densah bur at an rpm of 700 and a toque of 45 Ncm were used for implant placement. The primary stability of the implants placed using Densah was comparatively higher than that of implants placed using conventional drills. Postoperatively, an orthopantomogram (OPG) was taken to assess the implant position and adjacent bone. The patient was reviewed periodically, healing was satisfactory, and prosthetic rehabilitation was proceeded later.

## Introduction

The atrophic maxilla presents a challenging situation, making it difficult for a surgeon to plan rehabilitation in both surgical and prosthetic aspects [1]. Various factors contribute to this condition, and the major causes are pneumatization of the sinus, periodontal bone loss, and long-standing tooth loss. All these conditions lead to irreversible bone remodeling in a three-dimensional way that decreases the height and width of the bone. The conventional treatment methods include sinus lift, implant-retained overdenture, nasal floor augmentation, and bone grafts [2].

Huwais and Meyer were the first to introduce Densah burs in 2017 [3]. They documented that Densah burs facilitate osseodensification. This characteristic feature enhances the density and strength of the bone, thereby enabling increased primary stability of the implant [3]. These burs rotate in a counterclockwise direction that aids in densifying the bone rather than excavating it. This feature of the Densah bur makes it more reliable than conventional burs.

Here, we are presenting a case of a 63-year-old male patient who had a knife-edge atrophic posterior maxilla and underwent implant placement using Densah burs.

## Case presentation

A 63-year-old male patient presented to the Department of Oral and Maxillofacial Surgery with a chief complaint of missing teeth in the left upper back tooth region. History revealed that the patient underwent extraction at the same site three years ago. He underwent an implant rehabilitation at a private clinic on the opposite side of the same arch six months ago.

**Figure 1:**
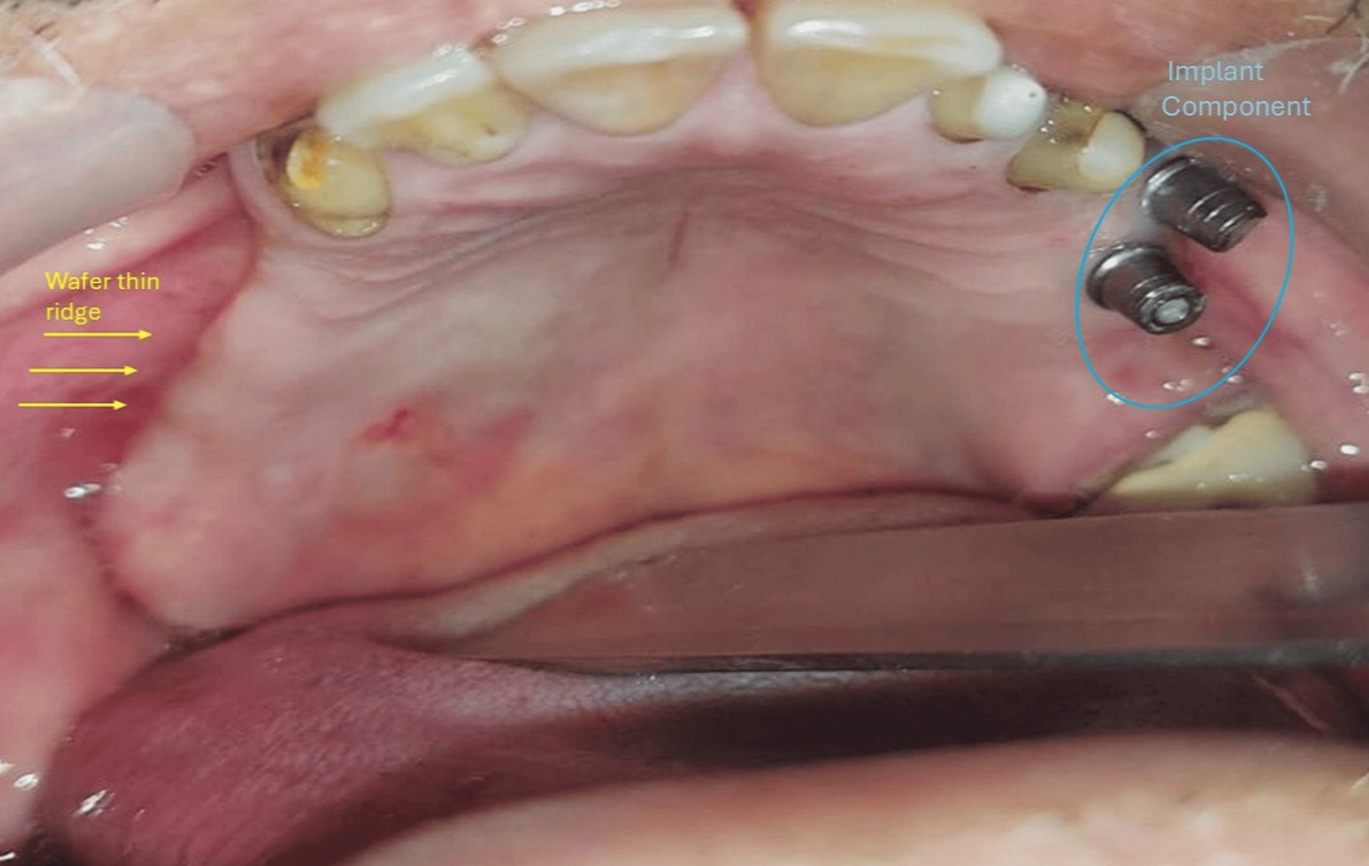
Intra-oral picture of the patient The yellow arrows indicate a wafer-thin, knife-edge ridge on the right side of the atrophic maxilla. The blue circle indicates the implant component seen on the second quadrant in regions 24 and 25.

The patient had no relevant medical history. On general examination, the patient is moderately built and nourished. Upon conducting an extra-oral examination, the facial appearance was apparently symmetrical, and the mouth was opening adequately. The intra-oral finding reveals missing teeth on the upper right posterior quadrant starting from regions 14 to 17, along with a knife-edge alveolar ridge and an implant component in relation to regions 24 and 25 (Figure 1).

**Figure 2:**
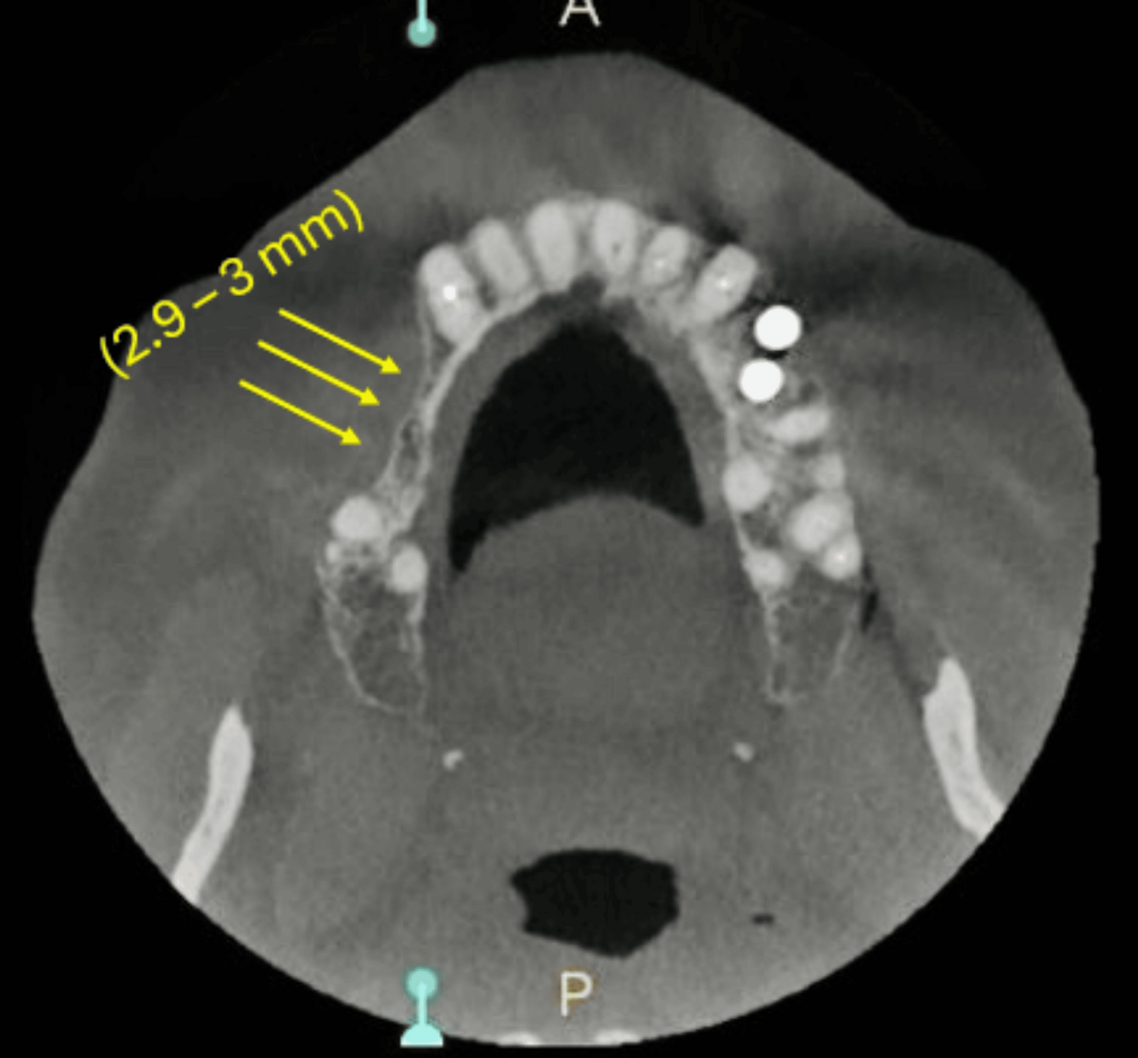
Axial view of CBCT The yellow arrows in the picture indicate the reduced buccolingual width (2.9-3 mm) of the alveolar bone. CBCT, cone beam computed tomography

**Figure 3:**
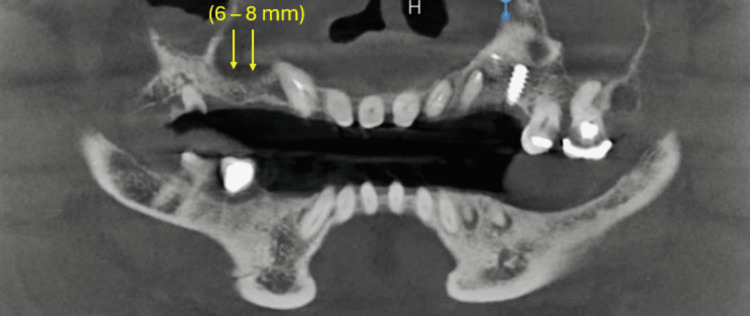
Coronal view of CBCT The yellow arrows indicate the reduced height (6-8 mm) of the alveolar ridge. CBCT, cone beam computed tomography

Routine blood investigations and cone beam computed tomography (CBCT) were carried out. CBCT revealed an overall reduction in buccolingual width, measuring 2.9-3 mm (Figure 2), and height, measuring 6-8 mm, of the right posterior maxillary region (Figure 3), suggestive of right posterior atrophic maxilla with D4 bone density (fine trabecular, Styrofoam posterior maxilla) [4].

Correlating clinical and radiological findings, the patient was suggested with multiple rehabilitation options. The initial treatment plan was ridge expansion with particulate bone graft placement, followed by implant placement after three months. However, the patient was unable to endure a lengthy wait for a prosthesis. Hence, to provide fixed rehabilitation in a short span of time, we used Densah bur to prepare the implant site, which has osseodensification potential.

**Figure 4:**
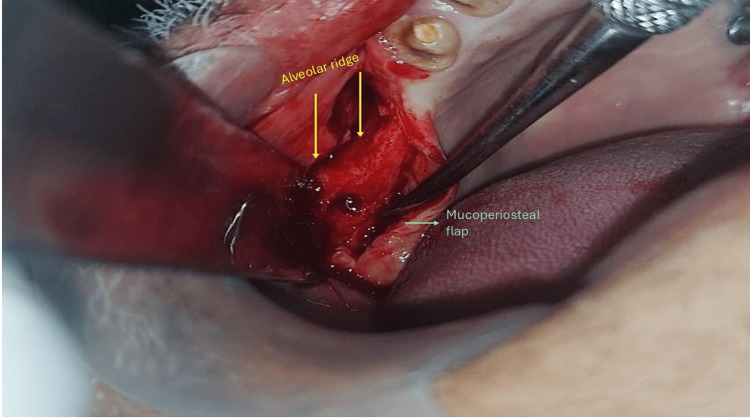
Intra-operative picture The mucoperiosteal flap is reflected, and the knife-edge alveolar ridge is exposed.

Under aseptic conditions, 2% lignocaine with adrenaline was used to give infiltration in the right vestibular area from regions 14 to 18 and the right greater palatine nerve block. Using the no. 15 blade, a crestal incision was made from regions 14 to 18 with a vertically released incision distal to region 13. A full-thickness periosteal flap is reflected. A wafer-thin, knife-edged alveolar ridge was visualized. Initially, we used conventional burs for pilot drilling followed by sequential drilling using Densah burs operating at an rpm of 700 in the counterclockwise direction (Figure 4). Three implants with a dimension of 3.5 × 10 mm have been placed. We assess primary stability using a torque wrench of 45 Ncm. Copious betadine saline irrigation was done. 3-0 Vicryl suture was utilized to achieve the primary closure, and hemostasis was achieved.

**Figure 5:**
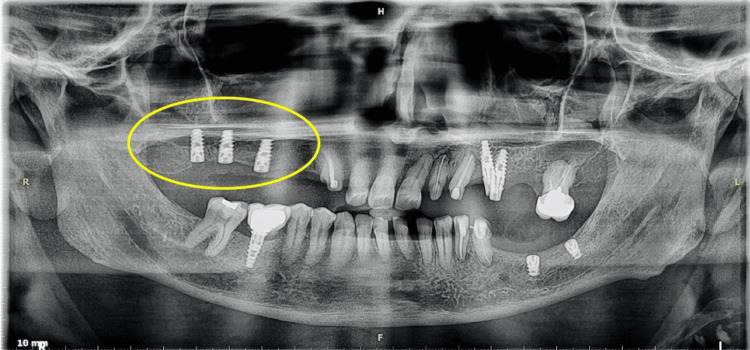
Postoperative OPG A three-implant prosthesis was placed with the help of Densah burs on the marked region. OPG, orthopantomogram

Postoperative healing was satisfactory. Orthopantomogram (OPG) was taken to assess the implant’s position (Figure 5). We kept the patient under periodic review for further prosthesis rehabilitation after three months.

## Discussion

The maxillary posterior region’s decreased bone height is caused by alveolar bone loss, sinus pneumatization, and prolonged edentulous conditions. This led to an atrophic maxilla. Rehabilitating the atrophic maxilla and achieving stability for the rehabilitated prosthesis can be challenging due to the reduced density and thinness of the bone in the atrophied region. Conventional methods employing burs rotate in a clockwise direction, resulting in the excavation of bone at the osteotomy site. The process of bone excavation thins out the maxillary and mandible. This method compromises the primary stability of the implant.

Therefore, in this case, a nonconventional method utilizing Densah burs was performed to treat atrophic maxilla. Densah burs were introduced as a novel biomechanical, minimally invasive technique. It aids in enhancing bone density through compaction grafting, thereby promoting osseous densification [5]. It has a non-cutting edge at 500-600 rpm. This non-cutting edge facilitates osseodensification. While drilling, the bone Densah burs rotate counterclockwise, autografting the bone in that site and making the bone dense.

Autografting helps in ridge expansion by densifying the crest. It also provides primary stability even in compromised bone anatomy. Densah burs increase the bone mineral density and also the amount of bone at the implant-placing surface [6].

The use of Densah burs for maxillary implant placement with transcrestal sinus augmentation yielded a favorable result [7]. Elghobashy et al. proved that radiographically, there was a significant increase in bone density at the implant site preparation [8]. Chen and Jeng hypothesized that the counter-clockwise rotating method has the ability to extend the ridge [9]. Al Ahmari concluded their study by stating that the osseodensification technique helped in achieving greater primary stability in low-density cases [10].

Similarly, in our case, we visualized the lateral and apical compaction of bone using Densah bur in a sequential manner. In this case, we were able to place an implant of ideal size with an alveolar bone width of 2.8 mm and a height of 8 mm and achieve optimal primary stability.

## Conclusions

To conclude, Densah burs play a pivotal role in achieving better results through the process of osseodensification. It minimized the overall treatment duration, even in the challenging maxilla.
